# Methemoglobinemia Caused by Topical Teething Preparation: A Case Report

**DOI:** 10.1100/tsw.2004.109

**Published:** 2004-07-15

**Authors:** Ran D. Balicer, Eliezer Kitai

**Affiliations:** ^1^ Department of Family Medicine, Sackler School of Medicine, Tel Aviv University, Sheba Medical Center, Building 130, IL-52621 Tel-Hashomer, Israel; ^2^ Leumit Health services, Israel

**Keywords:** methemoglobinemia, benzocaine, poisoning, Israel

## Abstract

Methemoglobinemia (MetHb) remains an uncommon, but potentially fatal disorder. Benzocaine (ethyl aminobenzoate), a topical anesthetic, has been reported to cause acquired MetHb when used during endoscopic or other ambulatory procedures. Reports of severe MetHb following benzocaine-containing preparations in the community, however, are very rare. We discuss this entity by describing an unusual case of severe MetHb in a 5-year-old child, caused by unattended self-use of a benzocaine-containing, pain-relief gel for teething. This case story illustrates the potential lethal risk of over-the-counter topical anesthetics for pediatric use. We review the risks of this potentially deadly disorder and the associated diagnostic challenges. Physicians not familiar with this rare complication may face diagnostic dilemmas, as its presentation is often nonspecific and rapid treatment is essential to prevent life-threatening complications.

## INTRODUCTION

Methemoglobinemia (MetHb) is an uncommon, but potentially fatal disorder. Benzocaine (ethyl aminobenzoate), a topical anesthetic, has been reported to cause acquired MetHb, most frequently when used during endoscopic and other ambulatory procedures. Reports of severe MetHb following benzocaine-containing preparations in the community are very rare[1]. We present this entity through an unusual case of severe MetHb in a 5-year-old child, caused by unattended self-use of a benzocaine-containing, pain-relief gel for teething.

## CASE STUDY

A previously healthy 5-year-old girl was brought unconscious to her family physician clinic. According to the parents, she appeared somewhat pale earlier that morning, but was feeling and acting well up until half an hour before arrival, when she suddenly began complaining of abdominal pain. Approximately 15 min later, she became lethargic and was rushed to the family clinic.

On admission to the clinic, the patient was not responsive, only to verbal stimuli with marked pallor with central cyanosis. Breathing was spontaneous, 16/min, with normal breath sounds bilaterally and blood oxygen saturation was 80%. Heart rate was 160/m with normal heart sounds and blood pressure 95/60 mmHg. Physical examination otherwise normal. Oxygen supplementation with a 100% mask was initiated and the patient regained consciousness within 5 min and was transferred to the closest medical center. On admission to the emergency room, blood oxygen saturation was as low as 67%, despite continuous 100% oxygen supplementation for nearly 20 min. Blood gases test showed PH of 7.45, bicarbonate levels of 24.7 mmol/l, PCO2 of 35 mmHg, PO2 of 315 mmHg, and base excess of +1.5 mmol/l. Blood count showed high leukocyte count (14,730/µl), while hemoglobin level and thrombocyte count were within normal range. Other lab tests including blood levels of sodium, potassium, urea, glucose, SGOT, alkaline phosphatase, lactic acid and CPK were all within normal range.

MetHb was suspected on the basis of the clinical and laboratory results, and a spectrophotometric test revealed methemoglobin levels as high as 64% (normal range: <1%), prompting immediate initiation of treatment with methylene blue. A single IV dose of 16 mg (1 mg/kg) methylene blue was given, followed by marked clinical improvement within a few hours. Blood methemoglobin levels were tested 2, 9, and 19 h after admission and gradually decreased to 6.1, 2.2, and 1.2%, respectively. The patient was discharged from the hospital within 24 h of admission.

Urine toxic screen indicated high concentrations of benzocaine. Further questioning of the mother revealed that the child had ingested an unknown amount of a topical pain-relief gel for teething, approximately 1 h before the onset of symptoms that morning. The gel, manufactured in the U.S., was purchased over the counter (OTC) and contained 7.5% benzocaine. The patient was treated with that same preparation several times during the first 2 years of her life without adverse effects.

## DISCUSSION

The clinical presentation of acquired MetHb may be confusing, particularly without obvious history of exposure to oxidizing agents. Generally, symptoms are worse with acute acquired MetHb than with hereditary types. MetHb refers to the oxidation of ferrous iron (Fe++) to ferric iron (Fe+++) within the hemoglobin molecule. This reaction impairs the ability of hemoglobin to transport oxygen and carbon dioxide, leading to tissue hypoxemia. The typical clinical signs and symptoms of MetHb by methemoglobin concentration are detailed in Table 1[2]. In our case, the patient fortunately responded promptly to methylene blue therapy and was released from hospital without sequelae, despite blood methemoglobin concentration reaching as high as 64% (a potentially fatal level).

Pulse oximeter may detect significant levels of methemoglobin as only mild to moderate oxygen desaturation and cannot be used to determine the actual percentage of MetHb in the blood. When MetHb levels reach 30–35%, the pulse oximeter reading becomes stable in the 82–86% range independent of actual methemoglobin levels[3]. The significant desaturation seen in our case (67%) can therefore not be solely attributed to the MetHb and may have been caused by reduced oxygenation during the period of unconsciousness.

Benzocaine-induced MetHb is an uncommon occurrence in routine clinical practice and since its first description in the literature in 1950, an overall count of 60 reported cases were noted by one recent study[4]. The vast majority of these reports refer to benzocaine-induced MetHb following endoscopic procedures, with an estimated incidence rate of 0.115% during transesophageal echocardiography[4]. The incidence of benzocaine-induced MetHb in the community is much more difficult to estimate. Lack of awareness and clinical misdiagnoses have likely led to an underestimation of its incidence and most available estimates are derived from case reports and reviews[5]. Studies indicate accidental ingestions of benzocaine-containing products only rarely lead to MetHb and this complication may be idiosyncratic and not dose related[4].

**TABLE 1**
**Clinical Manifestation of MetHb by MetHemoglobin Concentration**

| MetHemoglobin Concentration | % Total Hemoglobin* | Symptoms** |
|---|---|---|
| <1.5 g/dl | <10 | None |
| 1.5–3.0 g/dl | 10–20 | Cyanotic skin discoloration |
| 3.0–4.5 g/dl | 20–30 | Anxiety, lightheadedness, headache, tachycardia |
| 4.5–7.5 g/dl | 30–50 | Fatigue, confusion, dizziness, tachypnea, increased tachycardia |
| 7.5–10.5 g/dl | 50–70 | Coma, seizures, arrhythmias, acidosis |
| >10.5 g/dl | >70 | Death |

* Assumes hemoglobin = 15 g/dl. Patients with lower hemoglobin concentrations may experience more severe symptoms for a given percentage of MetHemoglobin level.
** Patients with underlying cardiac, pulmonary, or hematologic disease may experience more.

Reports of topical preparations as a cause of MetHb in community settings are scarce[1]. Although it remains an infrequently encountered entity, physicians not familiar with this disorder may face diagnostic dilemmas, as its presentation can be idiosyncratic and often nonspecific. Nevertheless, timely recognition of the condition is critical. Untreated, it can lead to serious complications and even death.

A recent study showed the main causes of OTC drug poisoning in children were parental lack of understanding the toxicity of OTC medications, lack of vigilance by parents or caregivers in the storage and administration of OTC medications, and lack of child-resistant closure designs for the medications[6]. In this case, the preparation indeed lacked child-resistant closure and the mother noted she believed pediatric OTC preparations to be invariably safe and therefore did not make an attempt to keep the tube out of her child's reach.

In conclusion, benzocaine-containing oral preparations may cause severe complications and should therefore be dispensed in child-resistant closure packs. Consideration should be given to increased counselling of consumers concerning the toxicity and safe storage of these OTC medications and the correct usage of child-resistant closure. Both health consumers and healthcare professionals should be made aware of this rare, but potentially fatal complication of topical anesthetic benzocaine-based oral preparations.

## BIOSKETCHES

**Ran D. Balicer, MD**, is affiliated with the Department of Family Medicine, Sackler School of Medicine, Tel Aviv University, Israel. E-mail: rbalicer@netvision.net.il

**Eliezer Kitai, MD**, is Associate Professor at the Department of Family Medicine, Sackler School of Medicine, Tel Aviv University, Israel, and heads the family medicine dept. in the Leumit Health services, Israel.E-mail: ekitaee@leumit.co.il
